# The Atlanta Society of Medicine

**Published:** 1895-01

**Authors:** 


### THE ATLANTA SOCIETY OF MEDICINE.

The indications are that the society has entered upon a career of increased usefulness and success. The society has abandoned its small room in the Fitten building, and through the courtesy of the Young Men's Christian Association, will hereafter meet in the auditorium of the latter building. This is a very important change, and cannot fail to be advantageous to the society's best interests. It is a fact that our meetings have not been properly attended, nor have the members shown the interest in the society which they should. We hope that this change will remedy all this, and that, with a more convenient and suitable place of meeting, every one will be on hand to contribute to the pleasure and profit of our bi-monthly meetings.

It is now proposed to establish a medical library for the society's use, and a committee has been appointed who will suggest plans for the same. This would be an exceedingly valuable feature of the society and it is hoped that successful plans will be reported and adopted. The library committee consists of Dr. W. S. Elkin, Dr. H. P. Cooper, Dr. W. A. Crow, Dr. C. D. Roy, and Dr. L. P. Stephens.

The officers of the society for the present year are: Dr. C. D. Hurt, President; Dr. R. R. Kime, Vice-President; Dr. W. L. Champion, Secretary; Dr. L. B. Grandy, Treasurer; and Dr. N. O. Harris, Corresponding Secretary.

